# Modeling and simulation of different and representative engineering problems using Network Simulation Method

**DOI:** 10.1371/journal.pone.0193828

**Published:** 2018-03-08

**Authors:** J. F. Sánchez-Pérez, F. Marín, J. L. Morales, M. Cánovas, F. Alhama

**Affiliations:** 1 Department of Applied Physics, Universidad Politécnica de Cartagena, Cartagena, Spain; 2 Department of Electrical Engineering, Universidad Politécnica de Cartagena, Cartagena, Spain; 3 Department of Structures and Construction, Universidad Politécnica de Cartagena, Cartagena, Spain; 4 Metallurgical and Mining Engineering Department, Universidad Católica del Norte, Antofagasta, Chile; Massachusetts Institute of Technology, UNITED STATES

## Abstract

Mathematical models simulating different and representative engineering problem, atomic dry friction, the moving front problems and elastic and solid mechanics are presented in the form of a set of non-linear, coupled or not coupled differential equations. For different parameters values that influence the solution, the problem is numerically solved by the network method, which provides all the variables of the problems. Although the model is extremely sensitive to the above parameters, no assumptions are considered as regards the linearization of the variables. The design of the models, which are run on standard electrical circuit simulation software, is explained in detail. The network model results are compared with common numerical methods or experimental data, published in the scientific literature, to show the reliability of the model.

## 1. Background

In the text of González-Fernández [1] can be found a detailed description of the fundamentals of the method and the first applications in different fields of science and engineering: electrochemical processes, transport through membranes, heat transfer, etc. After this date, the Network Simulation Method hereinafter (NSM), has been employed in new problems developing models not covered by a specific new text, so that the person concerned should refer to the specific scientific publications or doctoral theses in the research group ‘Simulation Networks’, from Universidad Politécnica de Cartagena (UPCT), or research groups working with this method at the Universities of Granada, Jaen and Murcia: [2–7]. In this regard it should be mentioned applications in the fields of fluid flow to transport (of mass or heat), inverse problem in heat transmission, magneto-hydrodynamic flow, mechanical vibrations, tribology, dry friction, membrane transport, elasticity, development of specific numerical calculation programs, etc.

NSM had already been successfully applied in various fields of engineering: heat transfer [7–11], electrochemical reactions [12,13], transport across membranes [14], inverse problems [15–18], ion transport, magneto-hydrodynamics [19], problems coupled flow and transport [17,20–22], and others [23–26]; all these works describe nonlinear transport processes. In addition, recently, it has been employed in mechanics of deformable solids [27], and dry friction at the atomic level.

On the other hand, there have been various programs that use the NSM as a tool for numerical calculation: PRODASIM for designing simple fins [28], PROCCA-09 for design and optimization of thermal problems [29], FATSIM-A for simulating flow fluids with solute transport problems [30], FAHET for simulation of flow fluids with heat transfer problems [31], EPSNET_10 for simulation problems elasticity [32] and OXIPSIS_12 for simulating corrosion problems [33].

The equivalence between the physical problem and the network model is that both are governed by the same differential equations in finite difference in space, covering both the elementary cell volume or as to the boundary conditions. However, time remains continuous variable in the design model.

The formal approach, which is the basis for the development of the problems, is the ‘Network Theory’ of Peusner [34], in which his ‘thermodynamics of networks’ is supported. Network models are for Peusner an accurate representation of the mathematic characteristic of the processes described. Thus, the variables which characterized of the problem must satisfy Kirchhoff’s laws and their relationships determine the corresponding circuit elements. However, in each individual process and once conjugate variables chosen, the information on which circuit elements involved in the network model and how they connect with each and other, is obtained from the mathematical model and not on considerations of physical type on the role playing these variables.

In summary, in network theory, the feasibility of a network model involves:

- The existence of an independent network of time
- The existence of a conserved quantity called flow associated with each branch, connecting nodes and obeys the Kirchhoff Current Law (KCL)
- The existence of a magnitude that satisfies criteria of uniqueness associated with each node, and obeys the Kirchhoff Voltage Law (KVL)

It is going to be showed different models of application of the method to different fields of engineering to be presented in the second section. In the third, the governing equations, and its transposition into network models in the fourth. Applications are going to be shown in the fifth section, concluding with the conclusions in the sixth.

## 2. Introduction

### 2.1 Dry friction between microscope tip and smooth surface at atomic-scale

This study will analyze the atomic scale dry friction phenomenon using models implemented by the network simulation method, namely the Frictional Force Microscope hereinafter (FFM), evolution of the Scanning Force Microscope hereinafter (SFM), increases research capacity of phenomena at the atomic scale, key issue for understanding the origin and nature of the fundamental laws of friction. The analysis of the behavior of these point contacts is done by a two-dimensional model of friction at the atomic scale. The response of the tip is the ‘stick-slip’ type [35].

After a detailed review of the literature a number of techniques and materials for analysis have been selected. The cases are:

- NaF (001): it is a surface with translational symmetry. In this case the FFM is used [36]
- Highly Oriented Pyrolytic Graphite hereinafter (HOPG) is well-defined structures sheets, often chemically stable. In this case an SFM and Lateral Friction Microscope hereinafter (LFM) are used [37]
- Graphite: the Atomic Friction Microscope hereinafter (AFM) is used [38]

### 2.2 Problems of moving front

In problems with phase transitions in matter, the Stefan problem is a particular kind of boundary problem, in which this phase boundary can move with time. The classical Stefan problem aims to describe the temperature distribution in a phase change of matter. These problems are known like ‘problems of moving front’ [39].

There are numerous problems of moving front, which mainly include the melting and solidification processes as well as the processes of oxidation at high temperature.

### 2.3 The elastic problem

Theory of Elasticity describes the response of a solid object to external forces [40]. The solution to this problem involves knowing the stresses, strains and displacements generated in the solid. The analysis results allow the engineer to make decisions on the validity or suitability of structures and/or machines. On the other hand, the elasticity theory equations are widely used in other fields of science and technology, such as the study of seismic wave propagation or biomechanical behavior.

## 3. The governing equations

### 3.1 Dry friction between atomic force microscope tip and smooth surface at atomic-scale

**Fig 1:**
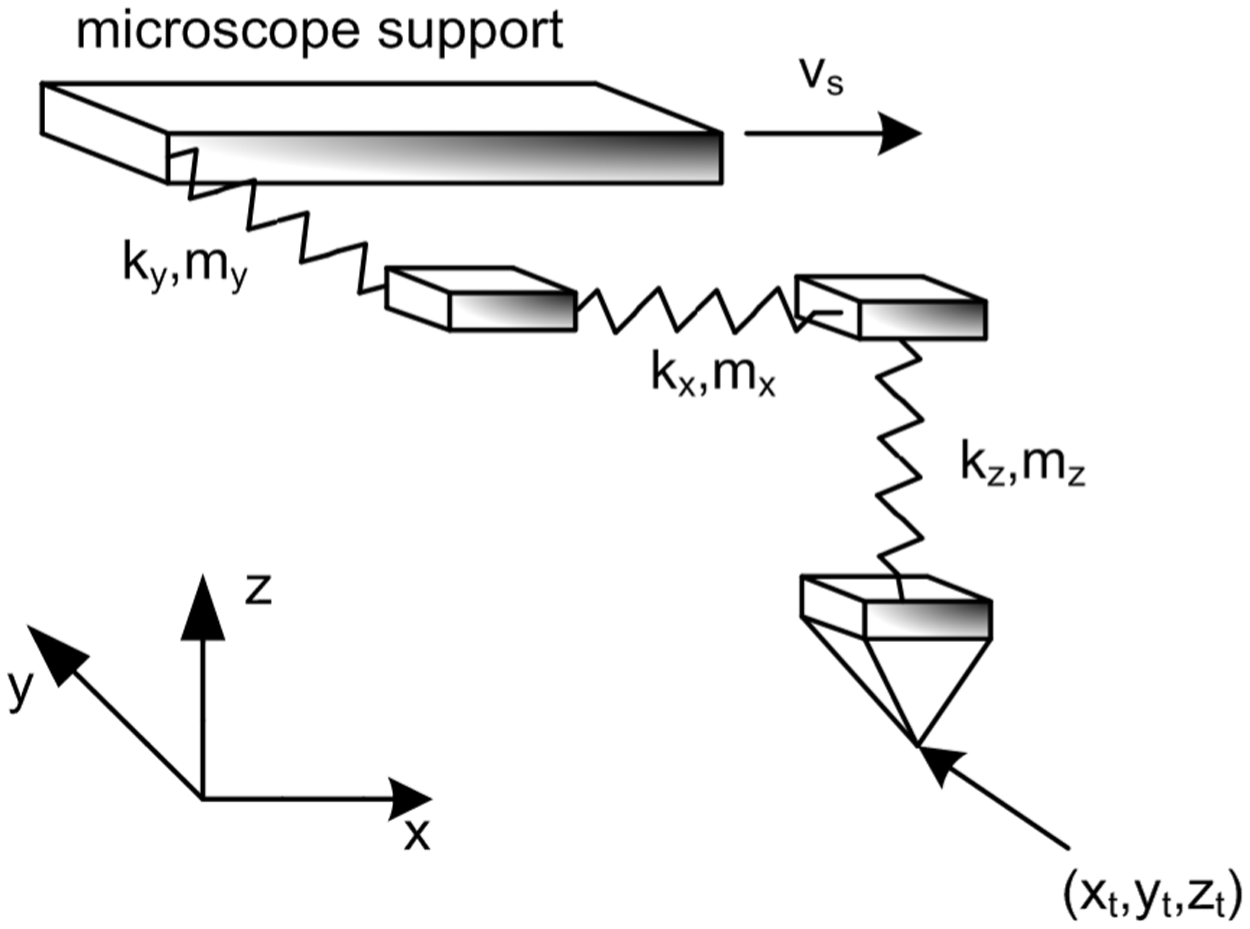
AFM, FFM and SFM tip model.

The AFM, SFM, FFM tip physical scheme on a surface model, Fig 1, considers one rigid sliding body connected by three springs (one for each spatial direction) to the mobile support. Each spring reflects the elastic interaction between the two surfaces during the contact [36,38]. The sliding body involves the interaction with the surface and the inertial effect.

Several forces act on the tip in x-direction: inertial force represented by ‘m·(*d*^2^x_t_/*d*t^2^)’, where x_t_ is the absolute displacement of the tip of mass m; elastic force from the spring, represented by the term ‘k_x_·(x_M_-x_t_)’; and damping force represented by the term ‘c_x_·(*d*x_t_/*d*t)’. The coefficients of these forces are constants. In addition, the surface-tip interaction force is built from a surface potential [36–38]. Thus, the NaF surface with FFM tip potential, Hölscher et al. [36] is:

$$V\left(x_{t},y_{t}\right)=V_{0}\cdot \text{cos}\left(\frac{2\pi }{a_{x}}\cdot x_{t}\right)\cdot \text{cos}\left(\frac{2\pi }{a_{y}}\cdot y_{t}\right)$$ (1)

where the potential amplitude, V_0_, is 1 eV, while a_x_ and a_y_, are 4.62 Å for both parameters. Hölscher et al. [37] use a slight modification of Eq (1) to study HOPG surface and a SFM tip:

$$V_{HOPG}\left(x_{t},y_{t}\right)=-V_{0}\cdot \left[2\cdot \text{cos}\left(\frac{2\pi }{a}\cdot x_{t}\right)\cdot \text{cos}\left(\frac{2\pi }{a\sqrt{3}}\cdot y_{t}\right)+\text{cos}\left(\frac{4\pi }{a\sqrt{3}}\cdot y_{t}\right)\right]$$ (2)

where V_0_ is 0.5 eV and the lattice constant, a, is 2.46 Å.

Sasaki et al. [38] used the Lennard-Jones potential to study the graphite and an AFM tip:

$$V_{TS}=\Sigma_{i}4\varepsilon \cdot \left[{\left(\frac{\sigma }{r_{0i}}\right)}^{12}-{\left(\frac{\sigma }{r_{0i}}\right)}^{6}\right]$$ (3)

where the strength of the potential, ε, is 0.87381·10^−2^ eV, and the value of the distance between the tip and the surface atom at which the potential is zero, σ, is 2.4945 Å. The tip and the i-th atom of the surface distance, r_0i_, is calculated from the i-th atom position inside the lattice, which is defined by its constant, 2.46 Å, and the nearest distance between 2 carbon atoms, 1.42 Å. Hölscher et al. employed the same potential with the same surface and microscope [41], but σ is equal to 3.4 Å.

As a consequence of applying the balance of the tip forces, Fig 1, the equations could be written:

$$\{\begin{array}{l} m\cdot {\ddot{x}}_{t}=k_{x}\cdot \left(x_{M}-x_{t}\right)-\frac{\partial V}{\partial x_{t}}-c_{x}\cdot {\dot{x}}_{t}\\ m\cdot {\ddot{y}}_{t}=k_{y}\cdot \left(y_{M}-y_{t}\right)-\frac{\partial V}{\partial y_{t}}-c_{y}\cdot {\dot{y}}_{t}\\ m\cdot {\ddot{z}}_{t}=k_{z}\cdot \left(z_{M}-z_{t}\right)-\frac{\partial V}{\partial z_{t}}-c_{z}\cdot {\dot{z}}_{t} \end{array}$$ (4)

The microscope movement is a set of trips in the x-direction.

### 3.2 Moving front problems

**Fig 2:**
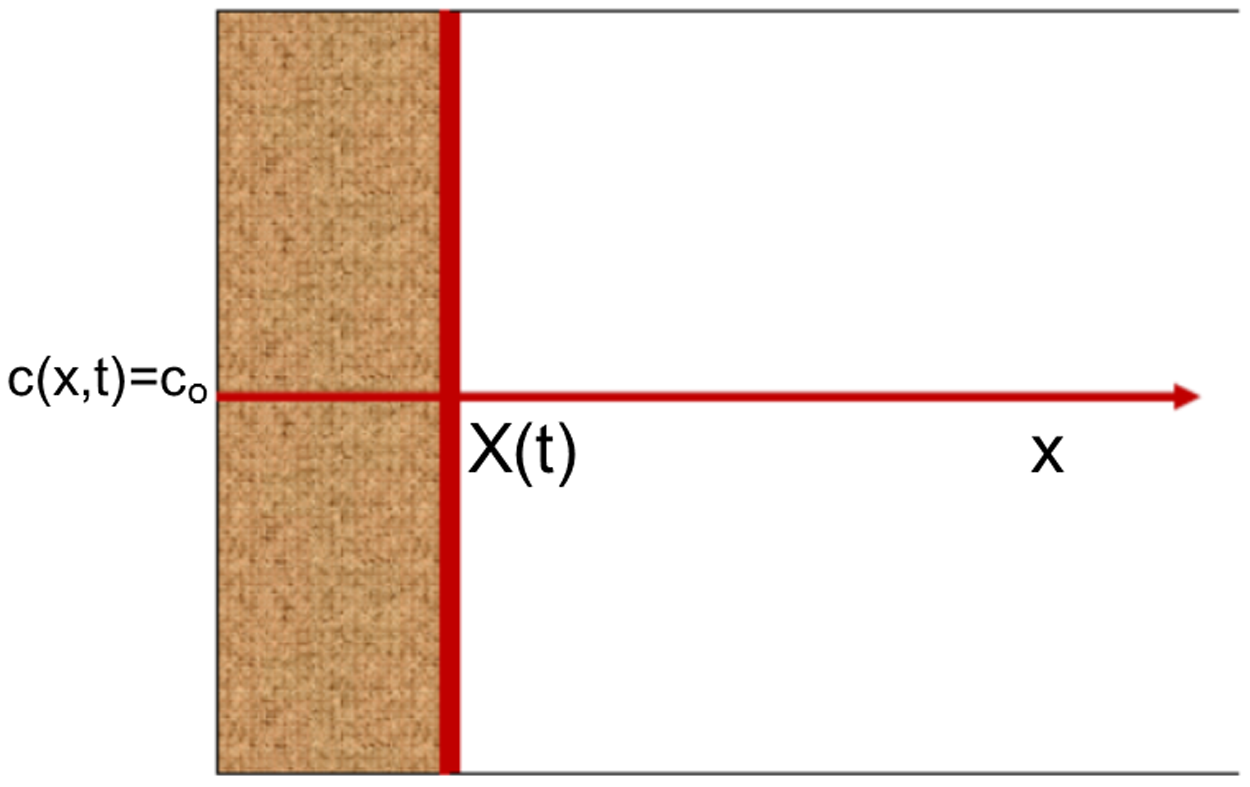
Scheme of areas within the metal, solid or liquid.

In moving front problems, there is increase, or decrease, of temperature, or a species, such as oxygen, increases its concentration. This depends on the process studied. The result is a new state or phase with different properties. Therefore, a new interface is generated. This interface is moved inside the structure producing the change of phase or state. Its position is represented as a distance function, Fig 2.

The problem considered has a semi-infinite structure, whose free surface, x = 0, is exposed to a constant concentration of oxygen or temperature, C_0_, [42,43]. These processes are almost instantaneous. Therefore, as soon as the temperature or the oxygen concentration reaches a determinate value, c_cr_, at one point, solidification or melting, oxidation interface will move to that point. The process described is a moving boundary problem, whose governing equations are:

$$D_{1}\frac{\partial^{2}{\text{c}}_{1}\left(\text{x},\text{t}\right)}{\partial {\text{x}}^{2}}=\frac{\partial {\text{c}}_{1}\left(\text{x},\text{t}\right)}{\partial \text{t}};\;0<x<X\left(t\right)$$ (5)

$$D_{2}\frac{\partial^{2}{\text{c}}_{2}\left(\text{x},\text{t}\right)}{\partial {\text{x}}^{2}}=\frac{\partial {\text{c}}_{2}\left(\text{x},\text{t}\right)}{\partial \text{t}};\;\text{X}\left(\text{t}\right)<x<\infty$$ (6)

where D_1_ and D_2_ are the diffusivities of each phase. The initial and boundary conditions are:

X(0)=0 (7)

$${\text{c}}_{2}\left(\text{x},0\right)=0,0<x<\infty$$ (8)

$${\text{c}}_{1}\left(0,\text{t}\right)={\text{c}}_{0},\;\text{t}>0$$ (9)

$${\text{c}}_{2}\left(\infty ,\text{t}\right)=0,\;\text{t}>0$$ (10)

The difference between flow values at the boundary between the two phases is responsible for the progress of the interface. Applying the conservation equation to concentrations [44,45], we have:

$$\phi_{1}\left(\text{X}\right)-\phi_{2}\left(\text{X}\right)=\Delta \text{c}\cdot \frac{\text{dX}}{\text{dt}}$$ (11)

where Δc is the oxygen concentration or temperature jump at the interface, and subscripts 1 and 2 refer to the oxide and metal phases or solid (or liquid and solid phase), respectively. Eq (11) is called the Stefan condition. This equation can be written as:

$$-D_{1}\frac{\partial {\text{c}}_{1}\left(\text{x},\text{t}\right)}{\partial \text{x}}=-D_{2}\frac{\partial {\text{c}}_{2}\left(\text{x},\text{t}\right)}{\partial \text{x}}+\Delta \text{c}\cdot \frac{\text{dX}}{\text{dt}},\;\text{t}>0$$ (12)

### 3.3 Governing equation for the elastic problem

**Fig 3:**
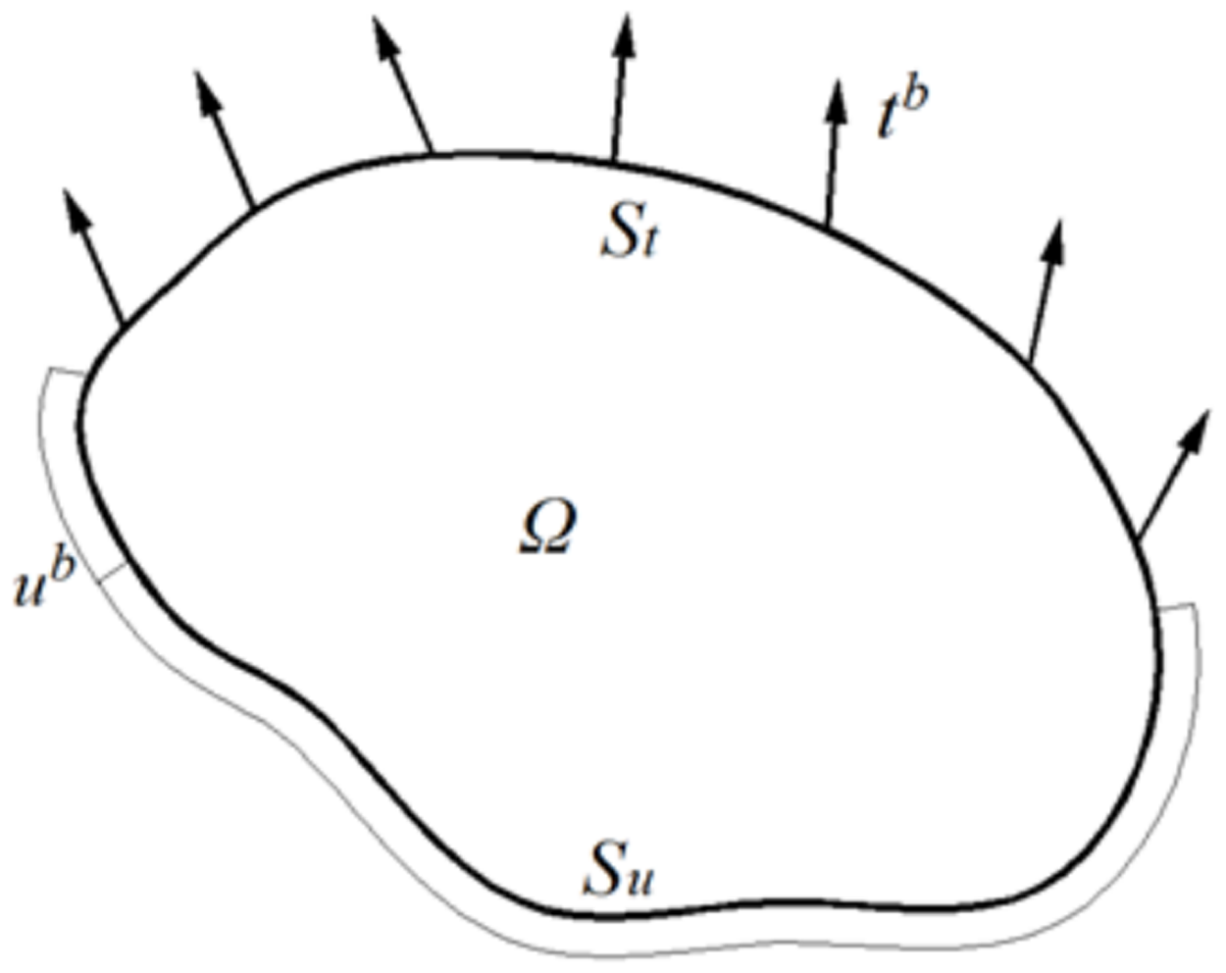
Elastic domain *Ω* under displacements *u*^*b*^ and tractions *t*^*b*^ boundary conditions defined over *S*_*u*_ and *S*_*t*_ respectively.

Navier’s equation [40], as the governing equation, represents the equilibrium forces in terms of displacements ***u*** for each point of the elastic body, Fig 3:*λ*

$$\mu \nabla^{2}u+\left(\lambda +\mu \right)\nabla \left(\nabla \cdot u\right)+f=0$$ (13)

where ***f*** is the volume force vector and *λ*, *μ* the material properties of the elastic body.

For 2D problems in Cartesian coordinates, Navier’s equations are reduced to two coupled differential equations:

$$\begin{array}{l} \mu \nabla^{2}u_{x}+\left(\lambda +\mu \right)\frac{\partial }{\partial x}\left(\frac{\partial u_{x}}{\partial x}+\frac{\partial u_{y}}{\partial y}\right)+f_{x}=0\\ \mu \nabla^{2}u_{y}+\left(\lambda +\mu \right)\frac{\partial }{\partial y}\left(\frac{\partial u_{x}}{\partial x}+\frac{\partial u_{y}}{\partial y}\right)+f_{y}=0 \end{array}\}$$ (14)

The required boundary conditions can be applied in displacements $u_{i}^{b}$ or tractions terms $t_{i}^{b}$, Fig 3. The first condition is directly imposed, but the second required a complex relation by coupling of the first partial derivatives of the main unknowns and the outer normal vector of the boundary ***n***:

$$\begin{array}{c} t_{x}^{b}=\left[\lambda \left(\frac{\partial u_{x}}{\partial x}+\frac{\partial u_{y}}{\partial y}\right)+2\mu \frac{\partial u_{x}}{\partial x}\right]n_{x}+\mu \left(\frac{\partial u_{x}}{\partial y}+\frac{\partial u_{y}}{\partial x}\right)n_{y}\\ t_{y}^{b}=\mu \left(\frac{\partial u_{x}}{\partial y}+\frac{\partial u_{y}}{\partial x}\right)n_{x}+\left[\lambda \left(\frac{\partial u_{x}}{\partial x}+\frac{\partial u_{y}}{\partial y}\right)+2\mu \frac{\partial u_{y}}{\partial y}\right]n_{y} \end{array}\}$$ (15)

Solved the elastic problem defined by Eq (14), the stress components can be evaluated from the displacement using the Lamé’s equations:

$$\begin{array}{l} \sigma_{xx}=\lambda \left(\frac{\partial u_{x}}{\partial x}+\frac{\partial u_{y}}{\partial y}\right)+2\mu \frac{\partial u_{x}}{\partial x}\\ \sigma_{yy}=\lambda \left(\frac{\partial u_{x}}{\partial x}+\frac{\partial u_{y}}{\partial y}\right)+2\mu \frac{\partial u_{y}}{\partial y}\\ \sigma_{xy}=\mu \left(\frac{\partial u_{x}}{\partial y}+\frac{\partial u_{y}}{\partial x}\right) \end{array}\}$$ (16)

## 4. Network models

### 4.1 Atomic force microscope simulation network models

The initial conditions related to displacements, x_M_-x_t_ = y_M_-y_t_ = z_M_-z_t_ = 0, and velocities, dx_t_/dt = dy_t_/dt = dz_t_/dt = 0, are inserted in the specifications of the capacitors initial conditions (initial voltage value) and of the coils (initial current value), respectively. The whole network model is now run employing PSpice [46,47].

The reliable network model design needs equivalence between the model and the process equations, including the initial conditions. Since Eq (4), the basic network model is corresponded with this equation, to which initial conditions must be added. Modeling basic rules are explained in González-Fernández [1]. The first is to select the equivalence between physical and electrical variables (different choices give rise to different networks). The following equivalence is established for the problem: x_t_ (tip displacement in the x-direction) ≡ q (electric charge in the network), or *dx*_*t*_/*d*t (tip velocity in the x-direction)≡*d*q/*d*t = i (electric current at the network). In the other directions, similar equivalences are applied.

Now, each addend of the Eq (4) is represented by an electric potential difference in a circuit element whose constitutive equation is analogous to the addend. Hence, the second Kirchhoff’s law application on a network equivalent to the equation allows us to solve the equation. The addend of Eq (4) ‘(*d*^2^x_t_/*dt*^2^)’ is correlated with an inductance whose constitutive equation is V_L_ = L(*d*i/*d*t) = L(*d*^2^q/*d*t^2^), with L = 1H. Equally, the same electrical element is used for the remainder directions. The non-linear terms cannot be implemented directly, and must be defined by controlled sources or additional circuits. In the event of voltage or current source, their output is defined by a routine as an arbitrary formula of the dependent variables. The most text books do not consider this classical electrical analogy in this way, becoming the NSM more interesting and efficient in the field of numerical computation.

**Fig 4:**
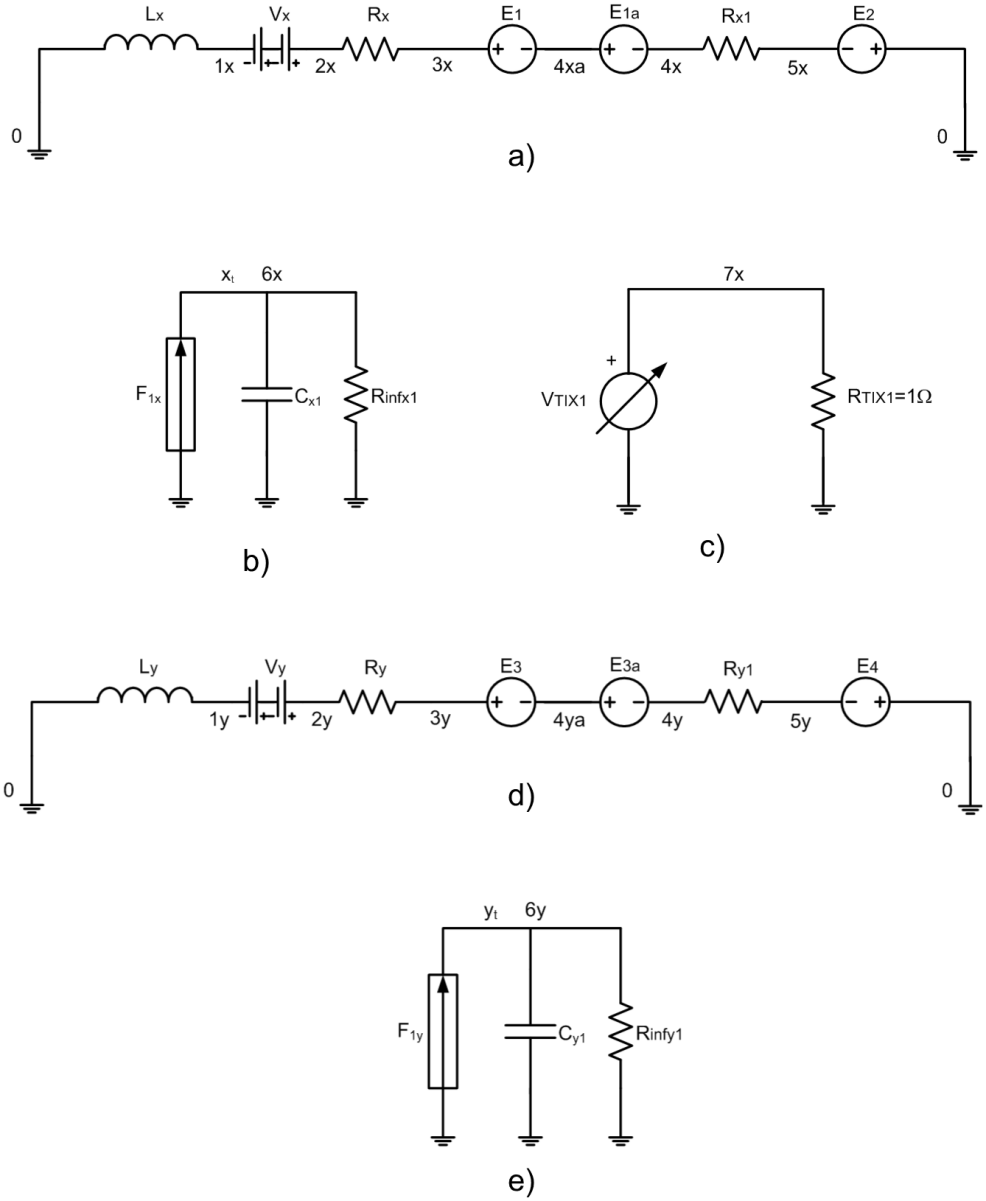
Network model of NaF with FFM and HOPG with SFM. a) and d) Main circuits, b) and e) secondary circuits to get x_t_ and y_t_, and c) secondary circuit to the time.

**Fig 5:**
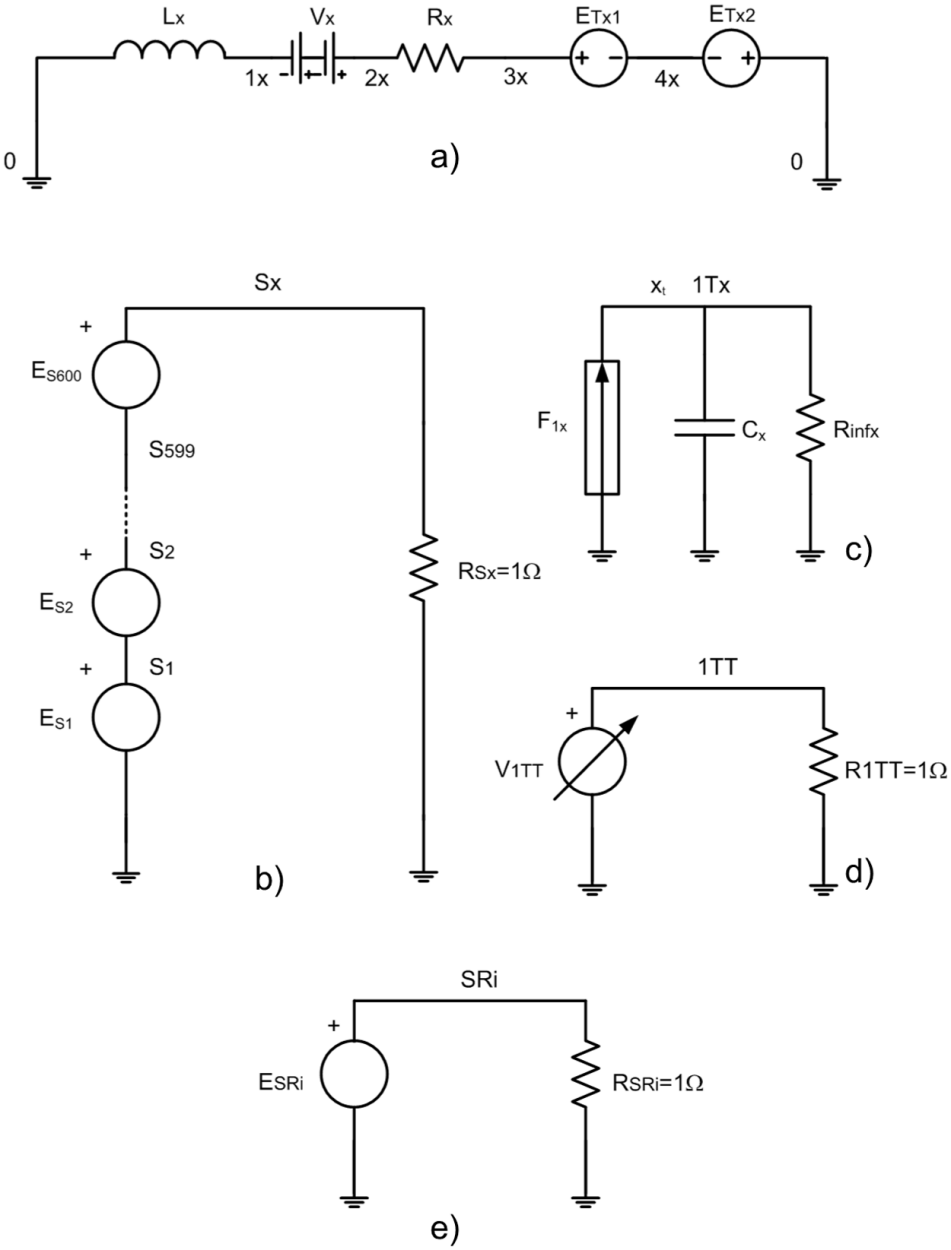
Network model of graphite with AFM. a) Main circuit, b) auxiliary circuit to get the force from Lennard-Jones potential, c) secondary circuit to get x_t_, d) secondary circuit to the time, and e) secondary circuit to get the AFM tip and carbon atom distance.

After the integration of ‘dx_t_/dt’, equivalent to the current in the main circuit, the term ‘x_t_’ is available. The integration is implemented using a secondary circuit in the models for NaF surface analyzed by FFM, Fig 4(b), for graphite surface analyzed by AFM, Fig 5(c). The terms ‘y_t_’and ‘z_t_’ are implemented in similar network models. The secondary circuit is implemented by a controlled source, F_1x_, which generates the current, ‘dx_t_/dt’, obtained from the voltage in an ammeter connected in series in the main circuit, V_x_. A capacitor with C_x1_ = C_y1_ = C_x_ = 1F is used to integrate the current of F_1x_, and the voltage in this element, V_Cx1_ = V_Cy1_ = V_Cx_ = Cx^-1^·∫(*d*x_t_/*d*t)*d*t, is simply the variable ‘x_t_’. A resistor with a very high value, R_INF_, improves the algorithm stability.

Once the variable ‘x_t_’ has been determined, the addend ‘k_x_·(x_M_-x_t_)’ in the model for NaF surface analyzed by a FFM, is implemented by a voltage from a controlled source, E_2_. For graphite surface analyzed by an AFM, another controlled source, E_Tx1_, provides the necessary voltage. The secondary circuit shown in Fig 4(c) provides the variable ‘x_M_’, the product of the constant velocity of the microscope support and time.

A pair of controlled sources, E_1_ and E_1a_, generates the voltage correspondent to ‘∂V/∂x_t_’, for NaF surface analyzed by a FFM. For graphite surface analyzed by an AFM, the controlled source, E_Tx2_, generates the voltage correspondent to potential gradient. The Lennard-Jones’ potential, represented in the secondary circuit of Fig 5(b), is selected. The addends associated to ∂V/∂y_t_ and ∂V/∂z_t_ are implemented in a similar way. The last one of the first equation in Eq (4) is equivalent to a resistor, characterized by ‘c_x_’, in the models for NaF surface analyzed by a FFM. For the remainder equations in Eq (4), a similar form is implemented.

The initial conditions corresponding to displacements, x_M_-x_t_ = y_M_-y_t_ = z_M_-z_t_ = 0, and velocities, dx_t_/dt = dy_t_/dt = dz_t_/dt = 0, are implemented by the initial conditions in capacitors and coils, respectively. PSpice [46,47] allows us to simulate the network model.

### 4.2 Moving front simulation network models

The details of the general rules of the NSM, with applications to different types of problems, are explained in González-Fernández and Alhama [1]. However, for a better interpretation the following steps are described for the design of the network model.

Firstly, the equivalence between the temperature or chemical concentration (oxygen concentration) and electrical variables: c ≡V_e_ (electric voltage) must be established.

**Fig 6:**
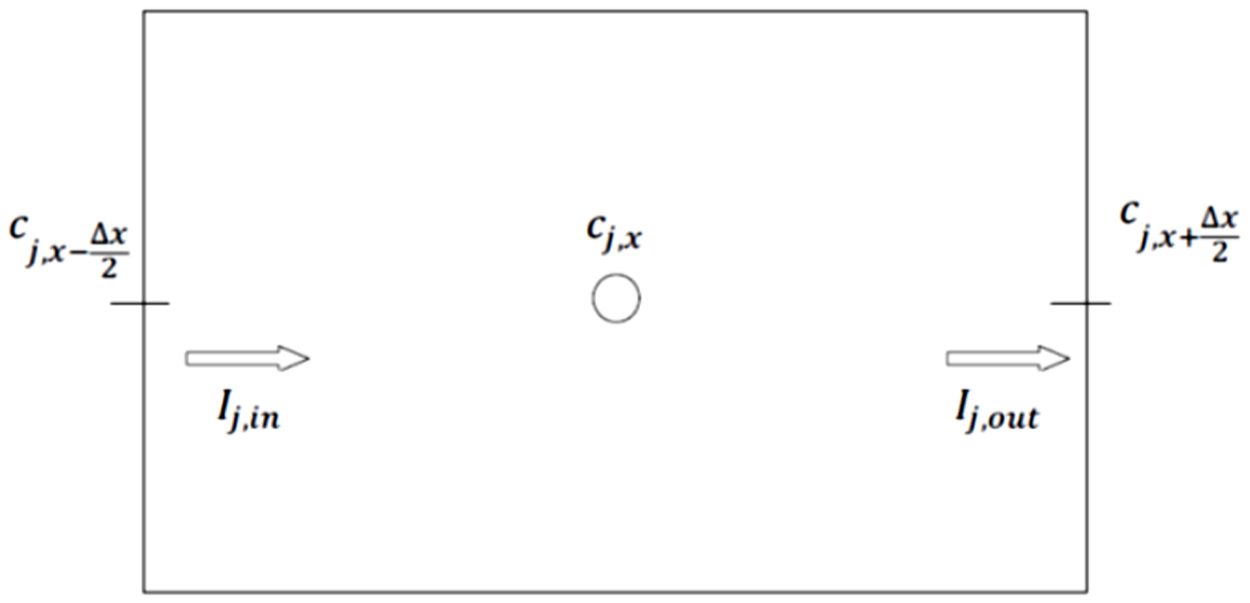
Nomenclature of the volume elements.

Secondly, it is considered that the process advances parallel to the surface so it is only necessary to consider a dimension. Therefore, the material is discretized in one-dimensional cells, dividing the spatial variable x into n volume elements, Fig 6. Thus, the terms in second derivatives of the above equations can be expressed in finite differences by

$$\frac{\partial {\text{c}}_{1}\left(\text{x},\text{t}\right)}{\partial \text{t}}=\frac{{\text{c}}_{1,\text{x}+\Delta \text{x}/2}-{\text{c}}_{1,\text{x}}}{\Delta {\text{x}}^{2}/2D_{1}}-\frac{{\text{c}}_{1,\text{x}}-{\text{c}}_{1,\text{x}-\Delta \text{x}/2}}{\Delta {\text{x}}^{2}/2D_{1}};0<x<X\left(\text{t}\right)$$ (17)

$$\frac{\partial {\text{c}}_{2}\left(\text{x},\text{t}\right)}{\partial \text{t}}=\frac{{\text{c}}_{2,\text{x}+\Delta \text{x}/2}-{\text{c}}_{2,\text{x}}}{\Delta {\text{x}}^{2}/2D_{2}}-\frac{{\text{c}}_{2,\text{x}}-{\text{c}}_{2,\text{x}-\Delta \text{x}/2}}{\Delta {\text{x}}^{2}/2D_{2}};\text{X}\left(\text{t}\right)<x<\infty$$ (18)

Thirdly, the right terms of the Eqs (8) and (9), dc_j_/dt are the currents that cross each capacitor, C_j_. The voltage across each capacitor, V_C,j_ = C_j_^-1^·∫(*d*θ_j_/*d*t)*d*t, is simply the variable c_j_ when C_j_ = 1F.

Fourthly, the Eq (10) gives the following differential finite difference equation:

$$-D_{1}\frac{{\text{c}}_{1,\text{x}+\Delta \text{x}/2}-{\text{c}}_{1,\text{x}-\Delta \text{x}/2}}{\Delta \text{x}}+D_{2}\frac{{\text{c}}_{2,\text{x}+\Delta \text{x}/2}-{\text{c}}_{2,\text{x}-\Delta \text{x}/2}}{\Delta \text{x}}-\Delta \text{c}\cdot \frac{\text{dX}}{\text{dt}}=0$$ (19)

The time derivative is discretized directly by the Spice Code Software tools [46,47], which automatically adjust the time increment to reach convergence more quickly. As mentioned with spatial discretization, the derivatives are associated with balances on the cell and not on each point of the mesh.

**Fig 7:**
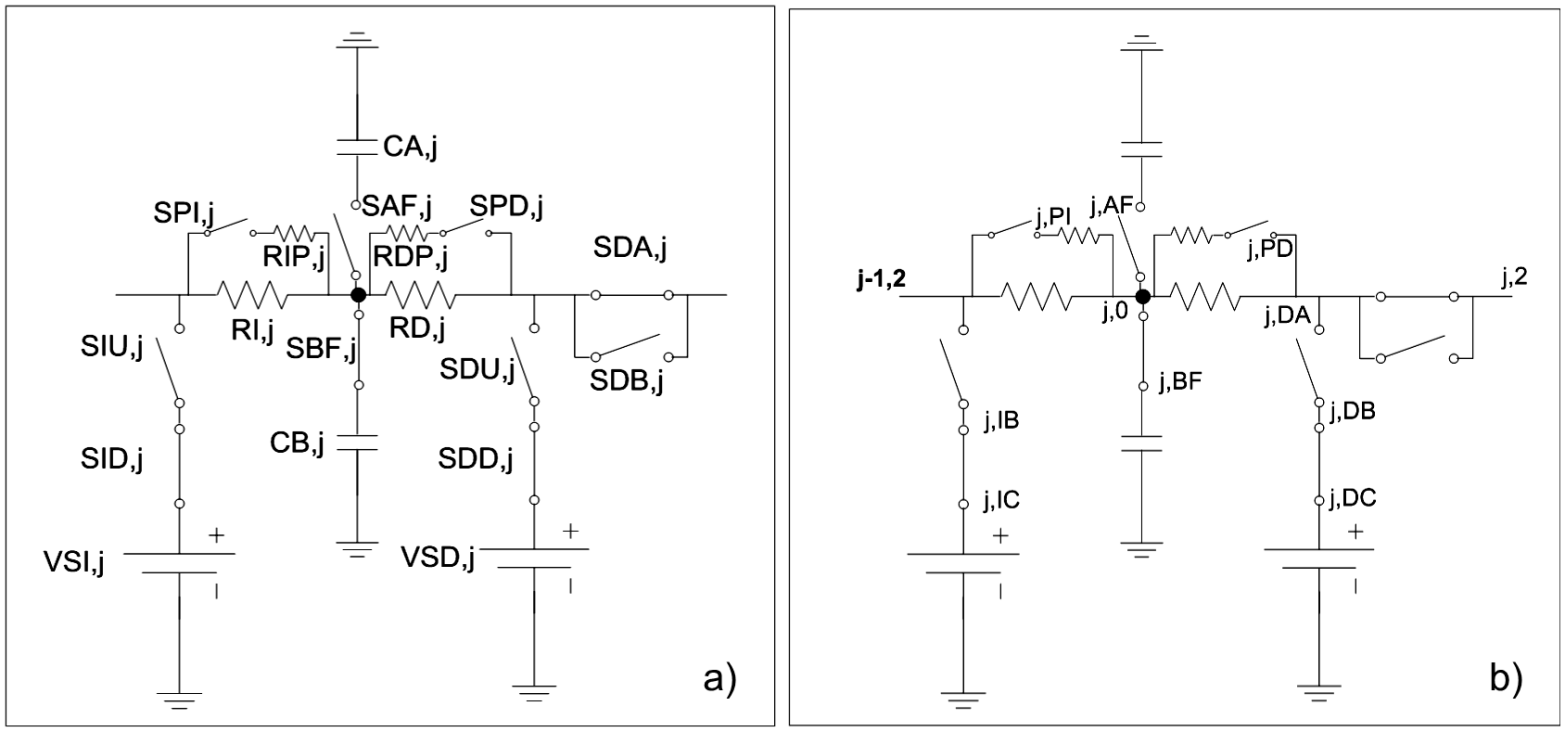
Equivalent electrical scheme of diffusion process in solid-liquid, liquid-solid or oxide-metal, system. a) circuit elements and b) nodes.

Fifthly, we proceed to the design of the network model. Fig 7 represents a cell inside the domain. In this circuit, each term, in Eqs (17) and (18), equal an electric current that is balanced with the current of another term in a common node.

The term on the left of the above equations, *d*c_j_/*d*t, is the current across the capacitor C_j_. The voltage in each capacitor, V_C,j_ = C_j_^-1^·∫(*d*θ_j_/*d*t)*d*t o V_C,y_ = C_y_^-1^·∫(*dy*/*d*t)*d*t is the variable c_j_ when C_j_ = 1F. Since there is a change of state in the cell, from metallic to oxidized, the capacitor used must have two different initial values, according to the state in which it is. In addition, with this software it is not possible to change the initial value of a capacitor, so it has been chosen to use two capacitors whose connection to the circuit is done through two switches controlled by voltage as indicated in Fig 7.

The last two linear terms of Eqs (17) and (18), I_j,in_ and I_j,out_, are represented as simple resistances, R_j,in_ and R_j,out_, respectively, since the equation of this electrical component is i_R_ = V_R_/R. The resistance is R_j,in_ = R_j,out_ = (Δx)^2^/2D_j_. As mentioned, a change of state occurs in the cell, so the resistors used must have two different values. Since it is not possible to change these values with this software, it has been chosen to introduce, again, two resistors in parallel for each addition. An open switch disables the resistance in parallel, leaving only the resistance representing the initial state. When the switch is closed, two parallel resistors whose combined effect represents the final state are enabled, Fig 7.

**Fig 8:**
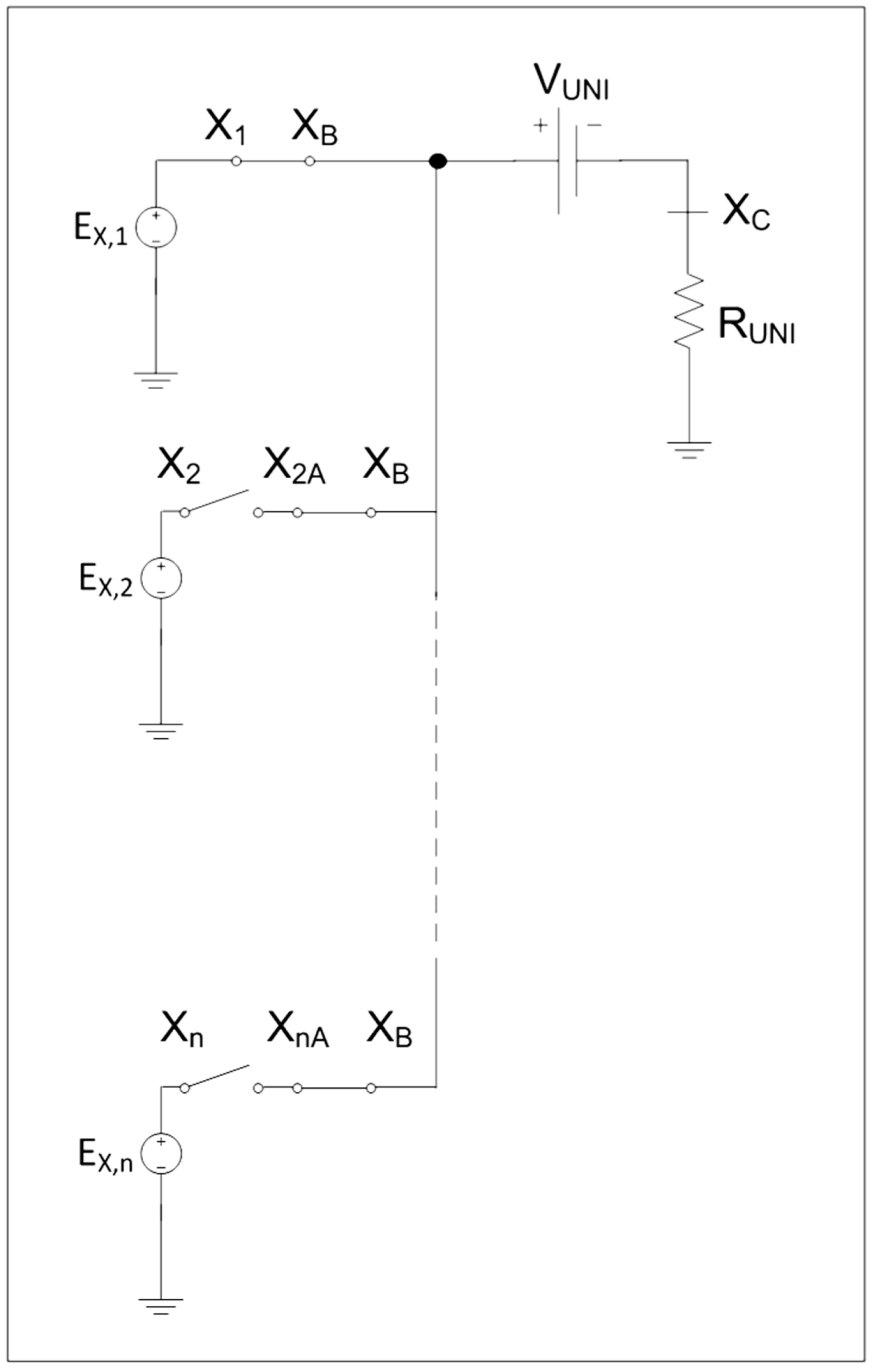
Network model of equilibrium equation.

Finally, each term in Eq (19) is considered a voltage equation and therefore a sequence of stresses that are introduced into the circuit by means of successive switches, allowing integrating the current position of the border, Fig 8 [44,45].

### 4.3 Network model for the elastic problem

According to the NSM rules [1], the resulting cell is derived from the spatial discretization of the governing equation, Eq (14), and the adequate electro-mechanical analogy. One circuit is resultant from each differential equation. Thus, the resulting cell involves two circuits related to the unknowns *u*_*x*_ and *u*_*y*_, respectively. Defining *C*_1_ = (*λ* + 2*μ*), *C*_2_ = *μ* and *C*_3_ = (*λ* + *μ*), governing equation can be written in a more compact form:

$$\begin{array}{r} C_{1}\frac{\partial^{2}u_{x}}{\partial x^{2}}+C_{2}\frac{\partial^{2}u_{x}}{\partial y^{2}}+\left[C_{3}\frac{\partial^{2}u_{y}}{\partial x\partial y}+f_{x}\right]=0\\ C_{2}\frac{\partial^{2}u_{y}}{\partial x^{2}}+C_{1}\frac{\partial^{2}u_{y}}{\partial y^{2}}+\left[C_{3}\frac{\partial^{2}u_{x}}{\partial x\partial y}+f_{y}\right]=0 \end{array}\}$$ (20)

**Fig 9:**
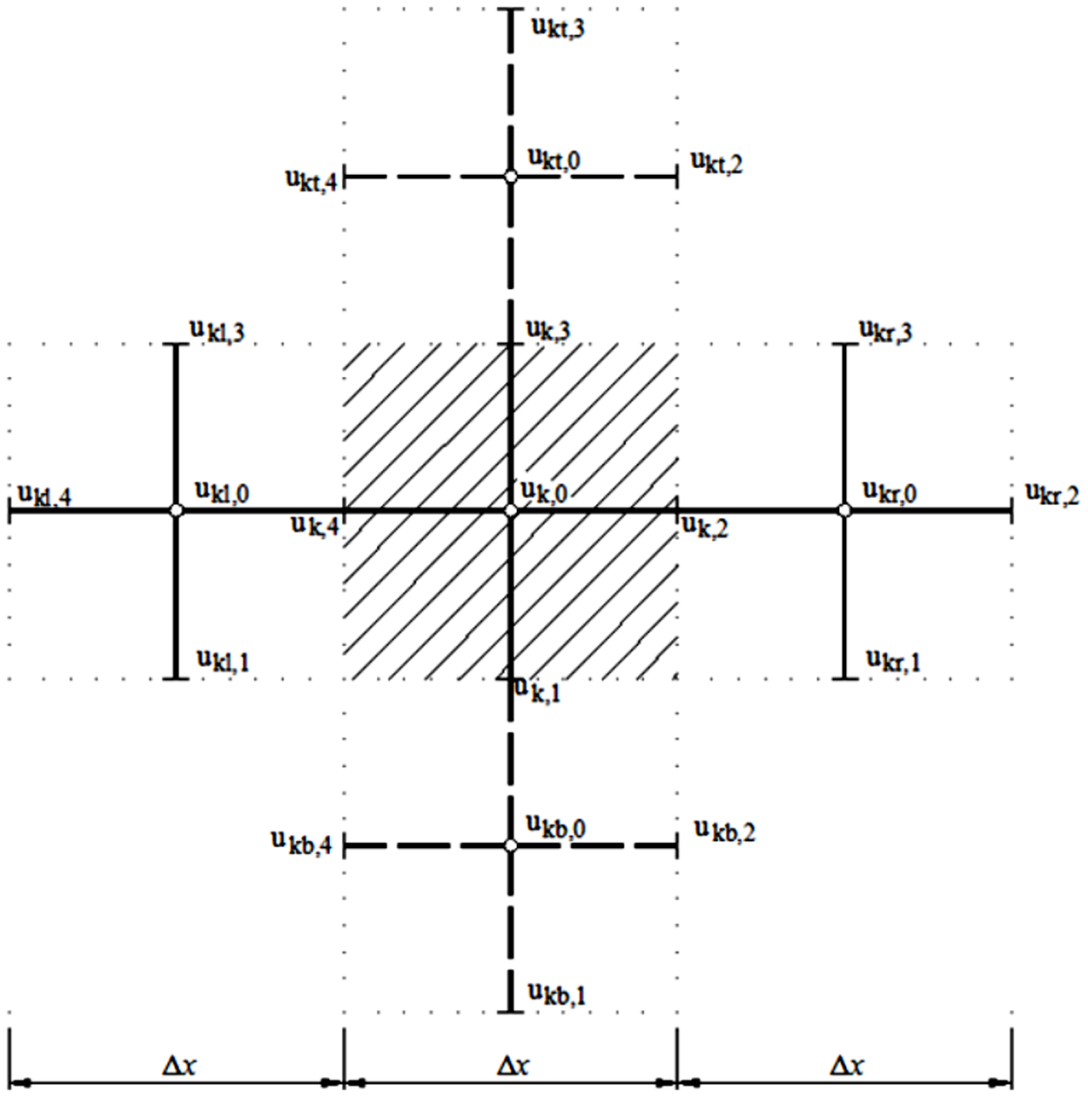
Mesh and notation.

Using the mesh and notation shown in Fig 9, each term in Eq (20) can be expressed in these finite differences:

∂2u∂x2|k,0≃uk,2−uk,0Δx2−uk,0−uk,4Δx2Δx=uk,2−2uk,0+uk,4Δx22=−(uk,0−uk,2Δx22+uk,0−uk,4Δx22)∂2u∂y2|k,0≃uk,3−uk,0Δy2−uk,0−uk,1Δy2Δy=uk,3−2uk,0+uk,1Δy22=−(uk,0−uk,3Δy22+uk,0−uk,1Δy22)∂2u∂x∂y|k,0≃ukt,2−ukt,4Δx−ukb,2−ukb,4Δx2Δy=ukt,2−ukt,4−ukb,2+ukb,42ΔxΔy} (21)

The resulting differences equations are:

uxk,0−uxk,2(Δx22)1C1+uxk,0−uxk,4(Δx22)1C1+uxk,0−uxk,3(Δy22)1C2+uxk,0−uxk,1(Δy22)1C2−[C3uykt,2−uykt,4−uykb,2+uykb,42ΔxΔy+fx]=0uyk,0−uyk,2(Δx22)1C2+uyk,0−uyk,4(Δx22)1C2+uyk,0−uyk,3(Δy22)1C1+uyk,0−uyk,1(Δy22)1C1−[C3uxkt,2−uxkt,4−uxkb,2+uxkb,42ΔxΔy+fy]=0} (22)

**Fig 10:**
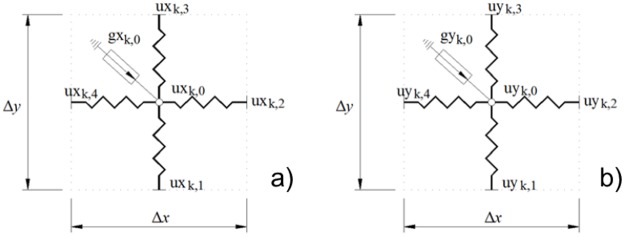
Cell of the network model. a) Circuit ‘ux’; b) Circuit ‘uy’.

Establishing the analogy between the mechanical displacement and electrical voltage, each equation shows a current balance. The resulting network model emerges from the electrical connection, in the whole domain N_*x*_×N_*y*_, among the cells shown in Fig 10.

The model presents two coupled circuits: circuit ‘ux’ in corresponding with the rectangular *u*_*x*_ displacement and one more for the *u*_*y*_ component. Each cell contains the following components for circuits: four resistors corresponding with the primary four addends in both equations; values of the resistors are expressed in the denominator. In other hand, coupled terms between equations and volume forces (last addends in brackets), are implemented by using a voltage-controlled voltage-source.

With regard to boundary conditions, while displacements are directly implemented by a constant voltage source, tractions, Eq (15), need the use of voltage-controlled voltage-sources.

## 5. Network models applications

### 5.1 Atomic-scale dry friction simulation: Atomic force microscopes network models

Network Simulation Method application of friction processes contained in the introduction is presented. For these processes, it is available partial results obtained by other authors with whom it is possible to verify the method. Furthermore, the effect of the parameters on the behavior of the proposed systems is discussed.

The NSM has been applied to studied surfaces and tips for which data are available: NaF and FFM tip [36], HOPG and SFM tip[37], and graphite and AFM tip [41].

#### 5.1.1 Friction force microscope on a sodium fluoride surface simulation

The tip characteristics, the surface size and the test conditions for the tests selected are:

- The interaction between the NaF surface and the FFM tip is defined m_x_ = m_y_ = 10^−8^ kg, c_x_ = c_y_ = 10^−3^ N·s/m and k_x_ = k_y_ = 10 N/m. The tip support velocity is 400 Å/s, the scan range of 20x20 Å and the scan angle of 0° are used in order to show the results [36]. The potential defined in Eq (1) is used in the model represented by Eq (4).
- The interaction between the HOPG surface and the SFM tip is defined by m_x_ = m_y_ = 10^−8^ kg, c_x_ = c_y_ = 10^−3^ N·s/m and k_x_ = k_y_ = 25 N/m. The tip support velocity is 400 Å/s, the scan range of 20x20 Å and the scan angle of 7° are used in order to show the results [36]. The potential defined in Eq (1) is used in the model represented by Eq (4). The studied surface, with a very high stiffness [37], is integrated by 271 hexagons, defined by six carbon atoms (600 altogether).
- The interaction between graphite surface and the AFM tip is defined by c_x_ = c_y_ = 0 N·s/m, k_x_ = k_y_ = 0.25–2.5 N/m, k_z_ = 0.25 N/m, and a punching of -6 Å. The scan range of 9.8x8.5 Å and the scan angle of 15° are used in order to show the results [38]. Besides, m_x_ = m_y_ = 10^−6^ kg and a tip support velocity of 10 Å/s, have been assumed in order to compare the results with Sasaki’s ones [38].

The potential functions defined in the following equations are used in Eq (4):

- Eq (1) in the NaF surface and the FFM tip model
- Eq (2) in the HOPG surface and the SFM tip model
- Eq (3) in the graphite surface and AFM tip model

In the last case, for graphite surface, the assumed model parameters are obtained using short computational time.

**Fig 11:**
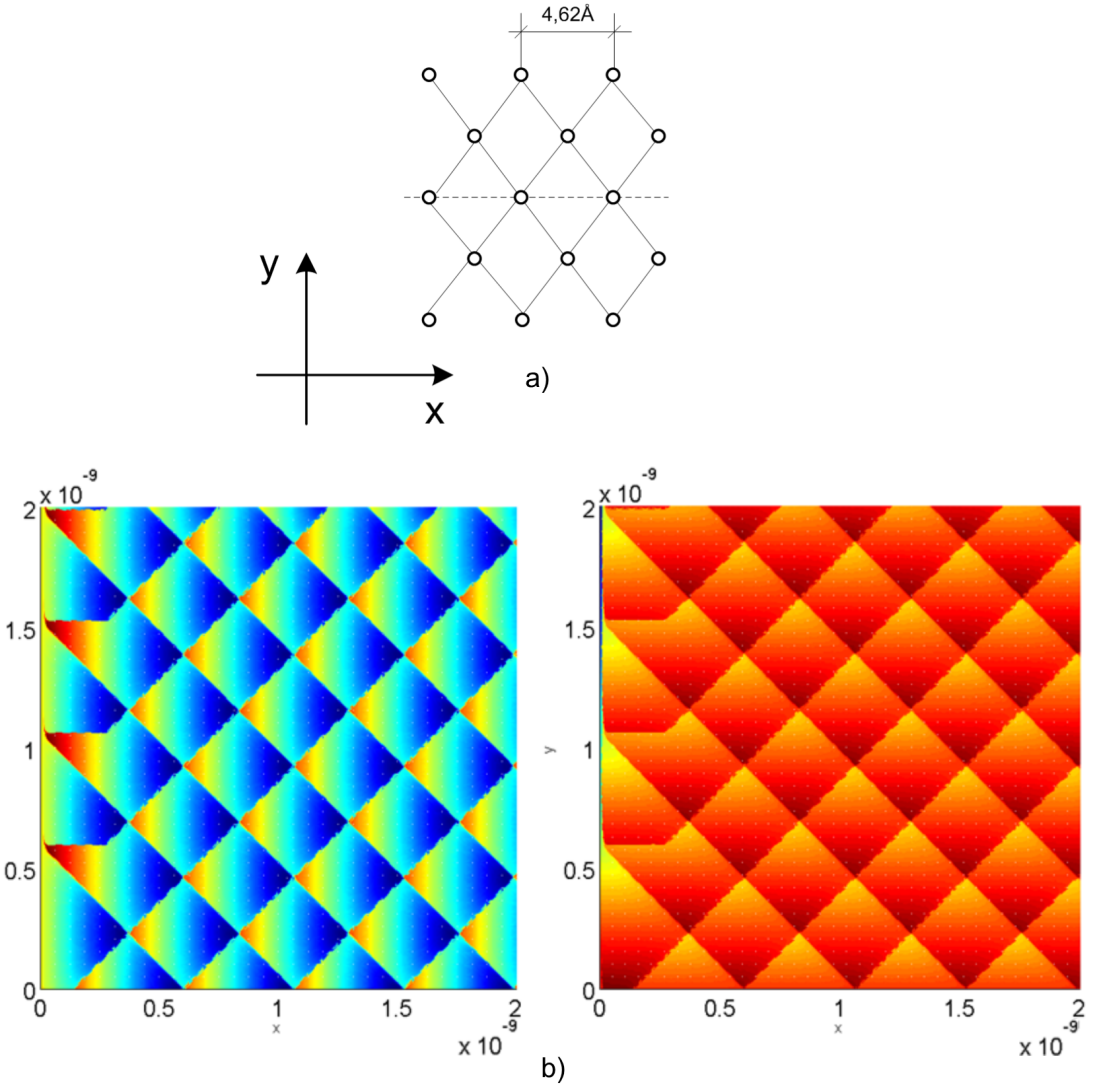
a) Position of the atoms in the NaF crystal net; b) Tip elastic force: F_x_ in the left and F_y_ in the right.

Fig 11(b) indicates the tip elastic force components, F_x_ and F_y_, in each NaF surface position, whose crystal net is presented in Fig 11(a). The reciprocation between both represented images allows us to handle this code as an image interpretation tool from the microscope. Thus, this code is capable to handle a lot of crystal nets, even with crystallographic defects, providing the appropriate elastic forces, which could be contrasted with those from the microscope. The simulation results correspond to those accomplished by Hölscher et al. [36].

#### 5.1.2 Atomic force microscope on a highly oriented pyrolytic graphite sample and on a graphite sample simulation

**Fig 12:**
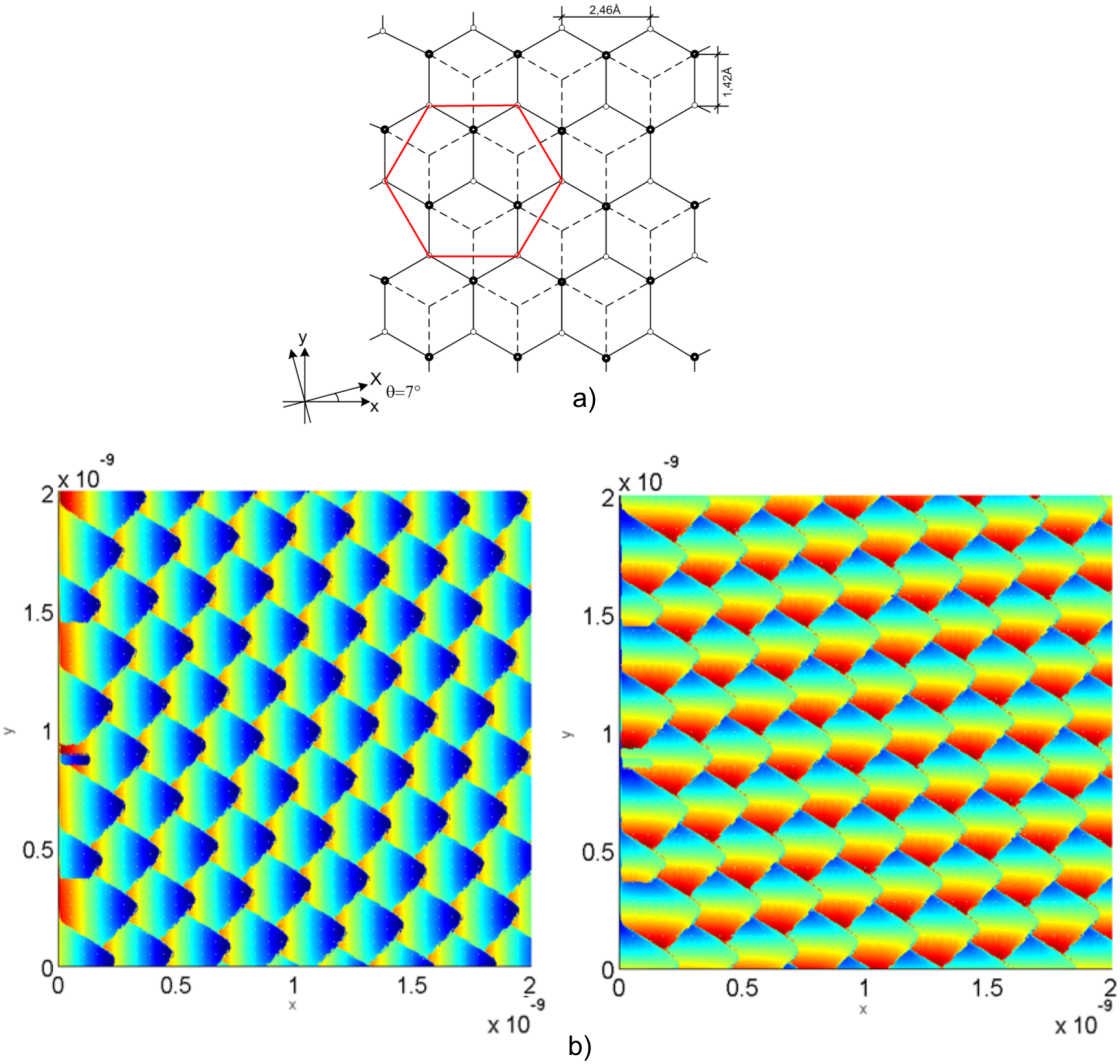
a) HOPG crystal net atoms position; b) Tip elastic force: F_x_ in the left and F_y_ in the right.

**Fig 13:**
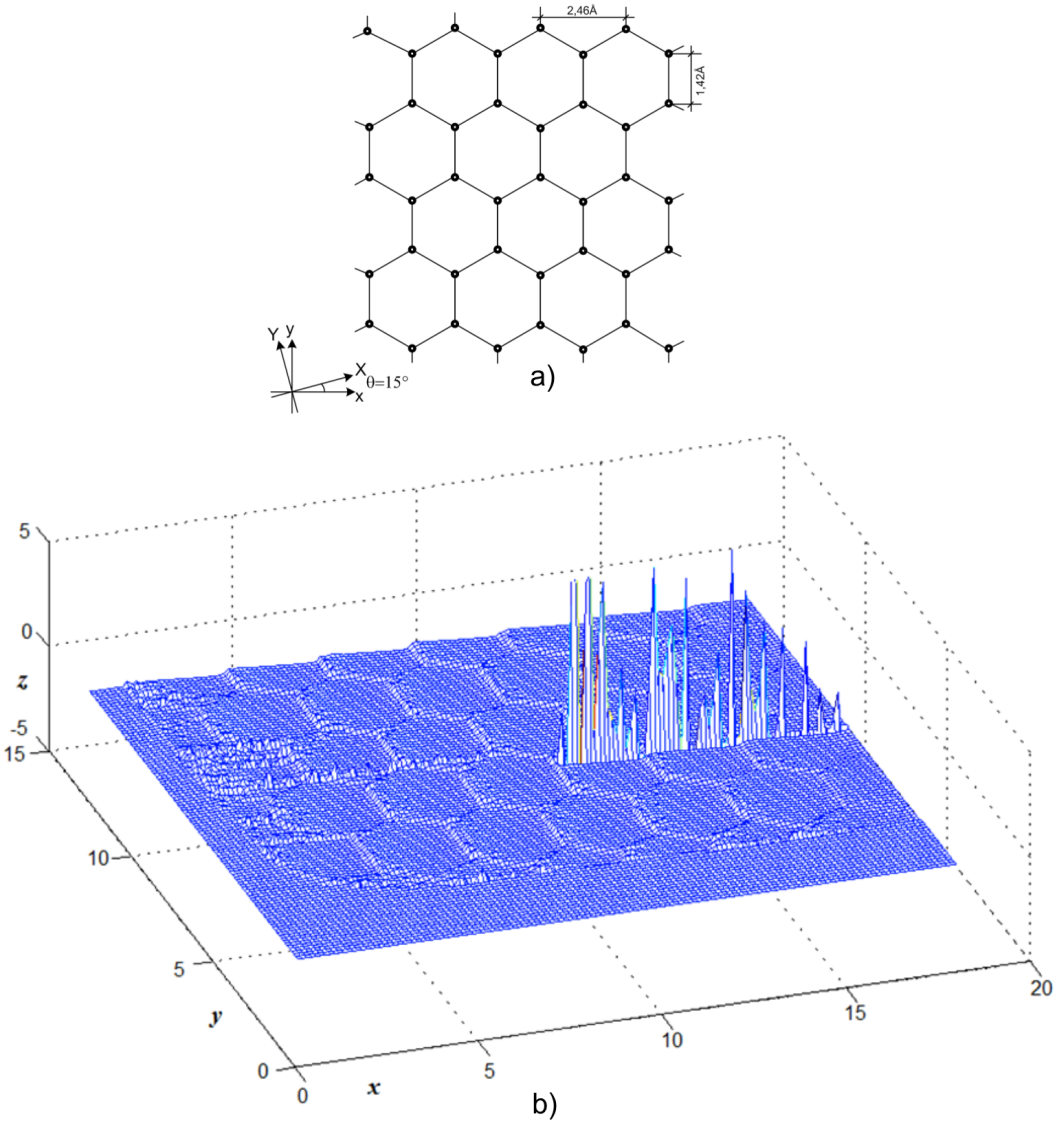
a) HOPG crystal net atoms position; b) Tip elastic force, F_x_.

Fig 12(b) indicates the tip elastic force components, Fx and Fy, in each HOPG surface position, whose crystal net is presented in Fig 12(a). The simulation results correspond to those provided by Hölscher et al. [37]. Besides, Fig 13(b) shows the elastic force x-direction, Fx, in each position of the graphite surface, Fig 13(a). In this case, a 3D plot is employed to depict the details better, specifically the stick-slip. The lines are plotted at different y-coordinates, separated by a fixed step. When one of these intersects the surface at a critical position, the instabilities appear, Fig 13(b). The simulation results correspond to those obtained by Sasaki et al. [38].

### 5.2 Moving corrosion fronts in titanium matrix composites

Lagoudas et al. [43] calculated the oxygen distribution throughout the oxide layer into the metal, assuming a rate position of the boundary between metal and oxide phases proportional to the square root of time. We solve this problem without making assumptions. In their analysis for SiC/Ti-15-3, used the values of diffusivity in the oxide, D_1_, and in the metal, D_2_, from the parameters of the Arrhenius equation, for temperatures between 600°C and 700°C, [39,48]. These parameters are shown in Table 1 [39,48–49].

**Table 1:** Coefficients to calculate the diffusivity.

Species	A (μm^2^/s)	E_a_ (cal/mol)
Metal (MMC)	7.37·10^6^	39960
Metal (Ti 99.9%)	17000	46062.1
Oxide	317.3	22756.2

Other authors, as Ariel et al. [49], estimate the diffusivity of oxygen in titanium 99.9% of purity, D_2_, using the parameters of the Arrhenius equation shown in the Table 1. We have used these parameters for modeling the oxidation of 99.9% pure titanium. Parameters of the oxide layer are the same as those used by Lagoudas *et al* [42,43]. The coefficients to calculate the diffusivity are defined in Table 1.

**Fig 14:**
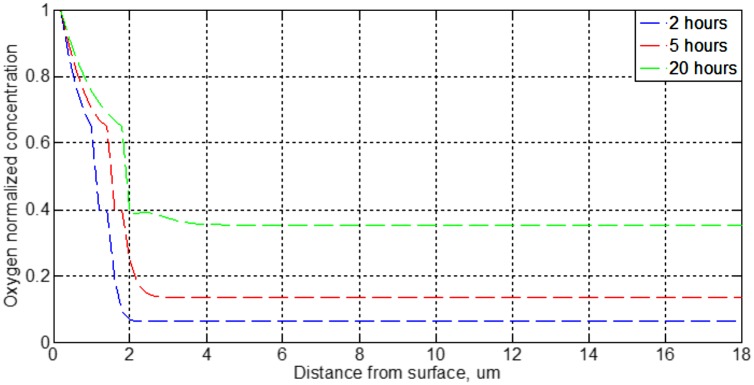
Distribution of oxygen concentration of the planar oxidation of Ti 99.9% for 2, 5 and 20 hours at 700°C.

Fig 14 shows the results for Ti 99.9% for different time intervals. As immediate conclusion, we can observe the progressive slowing down of the progress of oxidation, because of achieving an oxide thickness of 2 μm takes around 20 hours.

If Fig 14 is compared with previous work [45], it can be seen that the matrix SiC/Ti 15–3 is oxidized at a faster rate than titanium 99.9%. The explanation is the higher oxygen diffusivity of the first material. As shown, the second material takes nearly 20 hours to achieve an oxide thickness of 2 μm.

**Fig 15:**
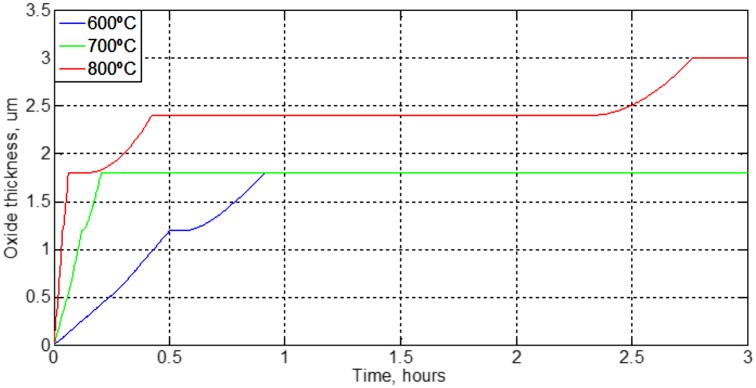
Evolution of interface at different temperatures of Ti 99.9%.

Fig 15 shows as the progress of the oxidation frontier for a given temperature is stopped when it reaches a certain position, a determinate oxide thickness. After a time interval, the frontier advance is restarted. Furthermore, as expected, an increase in temperature reduces this interval. This figure shows a radically different behavior of matrix SiC/Ti-15-3 [45]. The explanation for this different behavior is due to a titanium diffusivity value lower in three orders of magnitude as the matrix SiC/Ti-15-3.

Fig 15 shows better the slowdown of corrosion at different temperatures. As expected with increasing temperature is an increase of the oxidation rate. These results are the same as those established for titanium alloys by ASTM B861-10 [50] and ‘Asociación de Investigación de la Rama Metalmecánica de Valencia (AIMME)’ [51]. Their studies establish that the best alloys are those containing palladium and commercially pure titanium, 99.9%.

### 5.3 Cantilever beam subjected to shear loading

**Fig 16:**
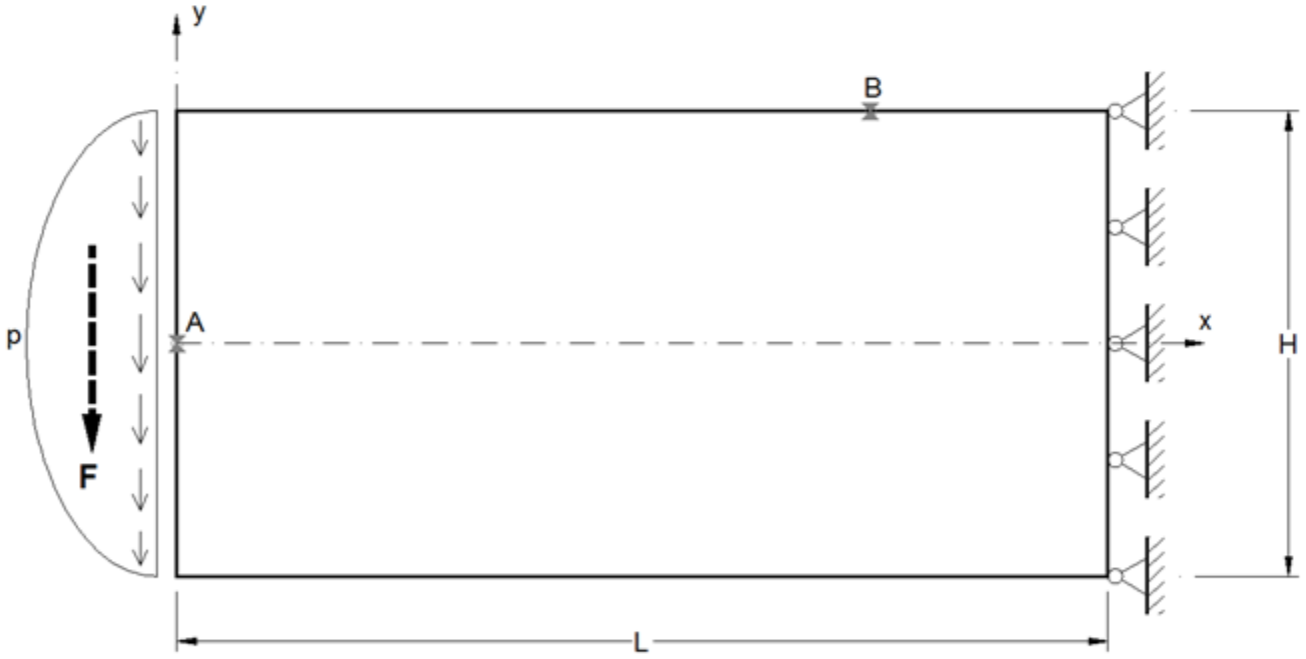
Cantilever beam subjected to shear loading.

To show the applicability of the NSM in the elasticity field bending of a cantilever is solved for a rectangular cross section, Fig 16, Morales et al. [52]. This plane stress problem has been studied by Timoshenko & Goodier [53] under ideally boundary condition. The resultant shear force at the free end F = 20·10^3^ N is applied following a parabolic distribution according with the elementary strength material theory (p = 30 MPa). For the right end the boundary condition zero-displacement is imposed. Other geometric and material parameters are L = 200 mm, H = 100 mm, thickness = 10 mm, λ = 121 GPa and μ = 80 GPa.

**Fig 17:**
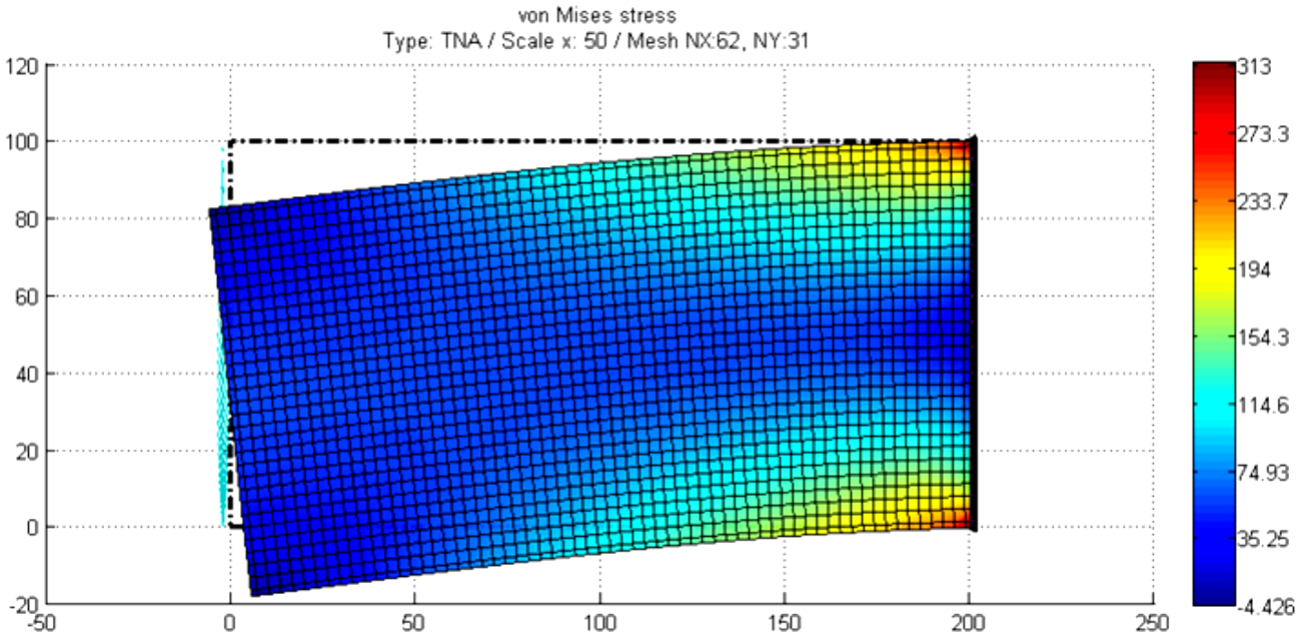
Von Mises stress in cantilever beam subjected to shear loading.

**Fig 18:**
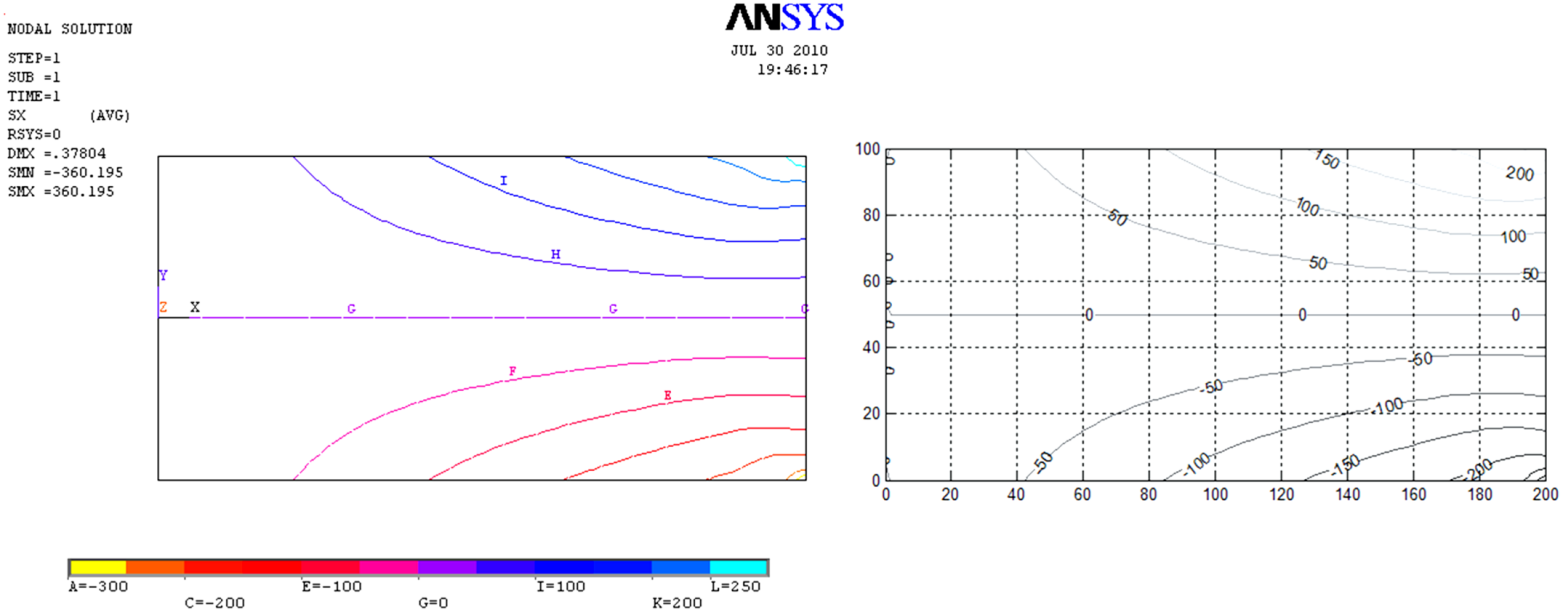
Stress isolines for the *σ*_*xx*_ component. Left: FEM solution. Right: NSM solution.

**Fig 19:**
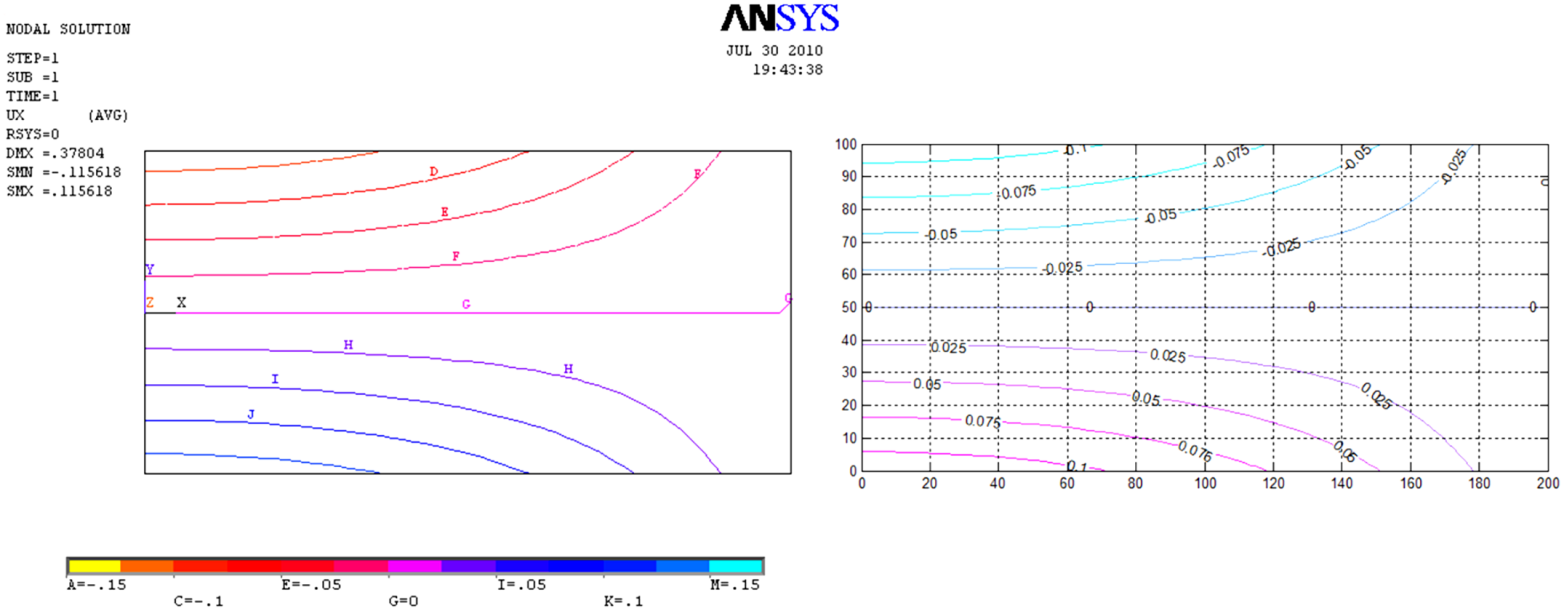
Displacement isolines *u*_*x*_. Left: FEM solution. Right: NSM solution.

**Fig 20:**
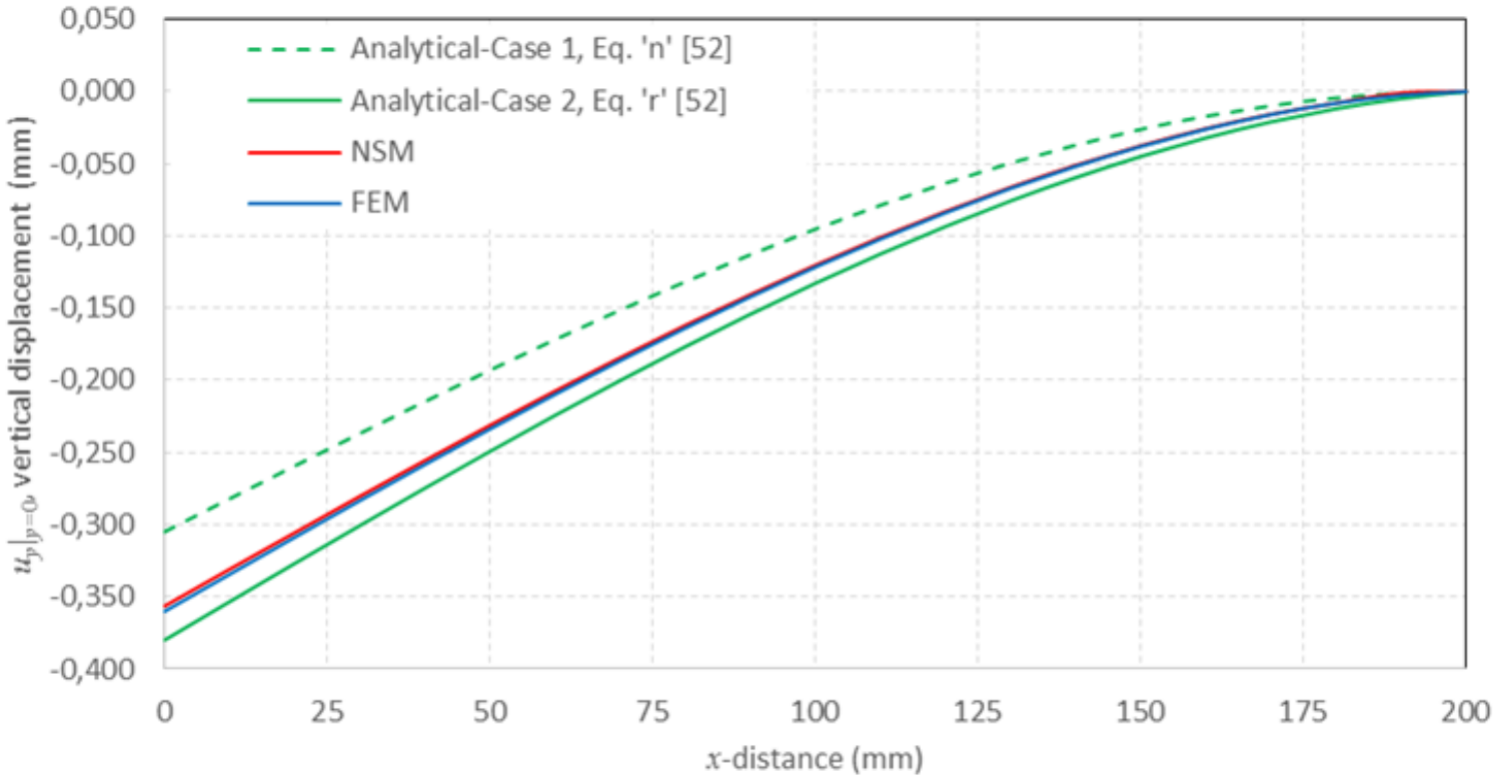
*u*_*y*_|_y = 0_ vertical displacement for analytical, NSM and FEM solutions at *y* = 0.

**Fig 21:**
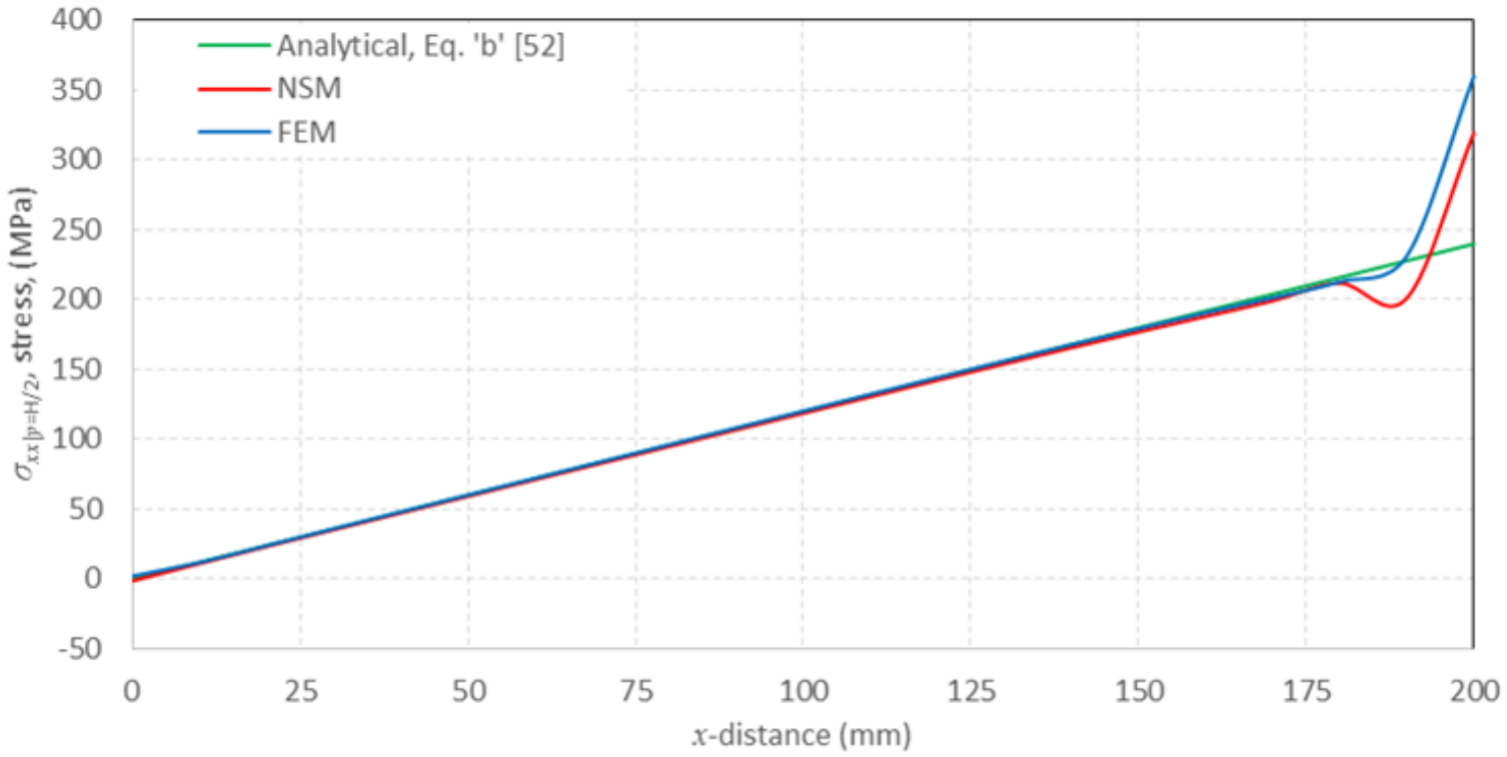
*σ*_*xx*_|_y = H/2_, normal stress component for analytical, NSM and FEM solutions at *y* = H/2.

Fig 17 presents the results of the network simulation in term of the von Mises stress over the deformed domain draw with a scale factor of 50. Next figures (Figs 18 and 19) exhibit a comparison between NSM and FEM [54] numerical simulations in terms of *u*_*x*_-displacement and *σ*_*xx*_-stress. Fig 20 shows the vertical displacements at *y* = 0 for the analytical solutions studied by Timoshenko and Goodier (two ideal cases, Eqs n and r [53]), and the numerical solutions obtained by means of NSM and FEM for the boundary condition depicted in Fig 16. The numerical solutions of the deflection curves are very closed and bounded by the two analytical and ideal cases. Fig 21 shows the *σ*_*xx*_-stress solutions for analytical and numerical methods for the upper edge line, *y* = H/2. These results are according to Saint-Venant’s principle, where only significant differences can be appreciated near to the right section. For a better comparison, Table 2 details numerical results in points A, in the origin, and B, at 3/4 of L. (Fig 16).

**Table 2:** Numerical results for FEM and NSM methods.

	FEM	NSM	Ratio	Deviation (%)
Point A: Deflection *u*_*y*,_ (mm)	-0.35995	-0.35620	0.9896	1.04
Point B: Stress *σ*_*xx*_, (MPa)	-179.45	-177.07	0.9867	1.33

## 6. Conclusions

Some numerical models based on the NSM have been designed and used—with negligible computing times in a suitable circuit simulation code—to successfully simulate different and representative engineering problems, such as dry friction in atomic scale, the moving front problems and elastic and solid mechanics, with no assumptions concerning the linearization of the governing equations. The influence of the problems’ main parameters is studied within a practical range of them. Comparison between the network model results and standard numerical methods or experimental data, which have been published in the scientific literature, confirms the reliability of the model.
